# Risk factors at five-year survival in grade 3 breast cancer: a retrospective observational study of the New Zealand population

**DOI:** 10.1186/s12889-021-12122-8

**Published:** 2021-11-06

**Authors:** Sharita Meharry, Robert Borotkanics, Reena Ramsaroop, Fabrice Merien

**Affiliations:** 1 School of Sciences, Faculty of Health and Environmental Sciences, Auckland University of Technology, Private Bag 92006, Auckland, 1142 New Zealand; 2 Faculty of Health and Environmental Sciences, Auckland University of Technology, Auckland, NZ New Zealand; 3 North Shore Hospital, Waitemata District Health Board, Surgical Pathology Unit, Auckland, NZ New Zealand

**Keywords:** Grade 3, Breast cancer, Prognostic factors, Survival

## Abstract

**Background:**

Breast cancer is the most common cancer in New Zealand, with approximately 3000 new registrations annually, affecting one in nine women and resulting in more than 600 deaths. This study analyzed data of patients with selected prognostic factors of Nottingham grade 3 tumors over a specified five-year period. The study aimed to identify factors that result in differential survival in the female, New Zealand population.

**Method:**

This is an observational, retrospective cohort study of prospectively collected data from New Zealand Breast Cancer Register. The selected period of 1st January 2011 to 31st, December 2015 allowed a consistent overlap for a national five-year data of grade 3 breast cancer in New Zealand. Mortality was carried out using univariate Fine-Gray competing risk statistical models.

**Results:**

This study showed that women in the older age group (> 70 years) had higher five-year mortality risk (HR: 1.7, 95% CI: 0.9–3.0, *p* = 0.053). Hormonal receptor analysis showed that ER positive, PR negative, and ER negative, PR negative subjects were at increased risk (HR = 3.5, 95% CI 2.3–5.4, *p* < 0.001) and (HR = 2.6, 95% CI, 1.8–3.9, *p <* 0.001) respectively. Molecular subtypes Triple Negative Breast Cancer and Luminal B subjects were at increased risk (HR = 3.0, 95% CI, 1.8–4.7, *p* < 0.001 and (HR = 3.3, 95% CI, 1.7–6.3, *p <* 0.001) respectively. HER2 enriched subjects were at a higher, but not significant, risk of five-year mortality compared to luminal A (HR = 1.6, 95% CI, 0.8–3.0, *p* = 0.10). NZ Europeans were at increased risk (HR = 1.7, 95% CI, 0.8–3.2, *p* = 0.11), with the highest Cumulative Incidence Function CIF, the largest proportion of HER2 enriched and TNBC across ethnicities.; however, Pacific Islanders experienced the highest HER2 CIF.

**Conclusion:**

The survival rates for grade 3 breast cancer vary across the selected prognostic factors and ethnicity. The results of this study make an initial contribution to the understanding of grade 3 breast cancer in the New Zealand population.

## Background

Breast cancer is the most frequently diagnosed cancer for women worldwide and the disease has a considerable impact on our society [1, 2]. New Zealand (NZ) is amongst the countries with the highest prevalence of breast cancer, with approximately 3000 new registrations per year, affecting one in nine women and resulting in more than 600 deaths annually [3, 4].

Epithelial breast tumors are graded according to the Nottingham criteria (based on morphology and proliferation of the breast tumor cells. This is a three-tier classification. Within this group of grade 3 breast cancer are tumors with varied biology and size thereby resulting in a heterogeneous group of tumors [1, 2, 5, 6]. This heterogeneity challenges the understanding of the pathology of these tumors. It is therefore of great value to understand the behavior of these often-aggressive cancers to establish an appropriate treatment and potentially improve the survival rate of women diagnosed with grade 3 breast cancer in New Zealand.

Previous studies using New Zealand Breast Cancer Register NZBCR have analyzed grade 3 breast cancers in conjunction with other grades and types but have not examined grade 3 breast cancer as a stand-alone group. This study will be unique in detailing chosen prognostic factors within grade 3 breast cancer in the New Zealand population.

The population of New Zealand is made up of very diverse ethnic groups. The presence of the largest population of Pacific Islanders (8.1%) outside of the Pacific Islands makes it a unique population. However, the largest ethnic group by far are Europeans followed by Maori [7–10].

Within this diverse ethnicity, breast cancer can be assessed for variable presentation, biology, and survival. This provides a comparison with global literature and a unique report on breast cancer mortality using the New Zealand Breast Cancer Register (NZBCR).

The results should make an initial contribution to the understanding of this selected heterogenous high-grade group.

## Methods

### Study design

**Fig. 1:**
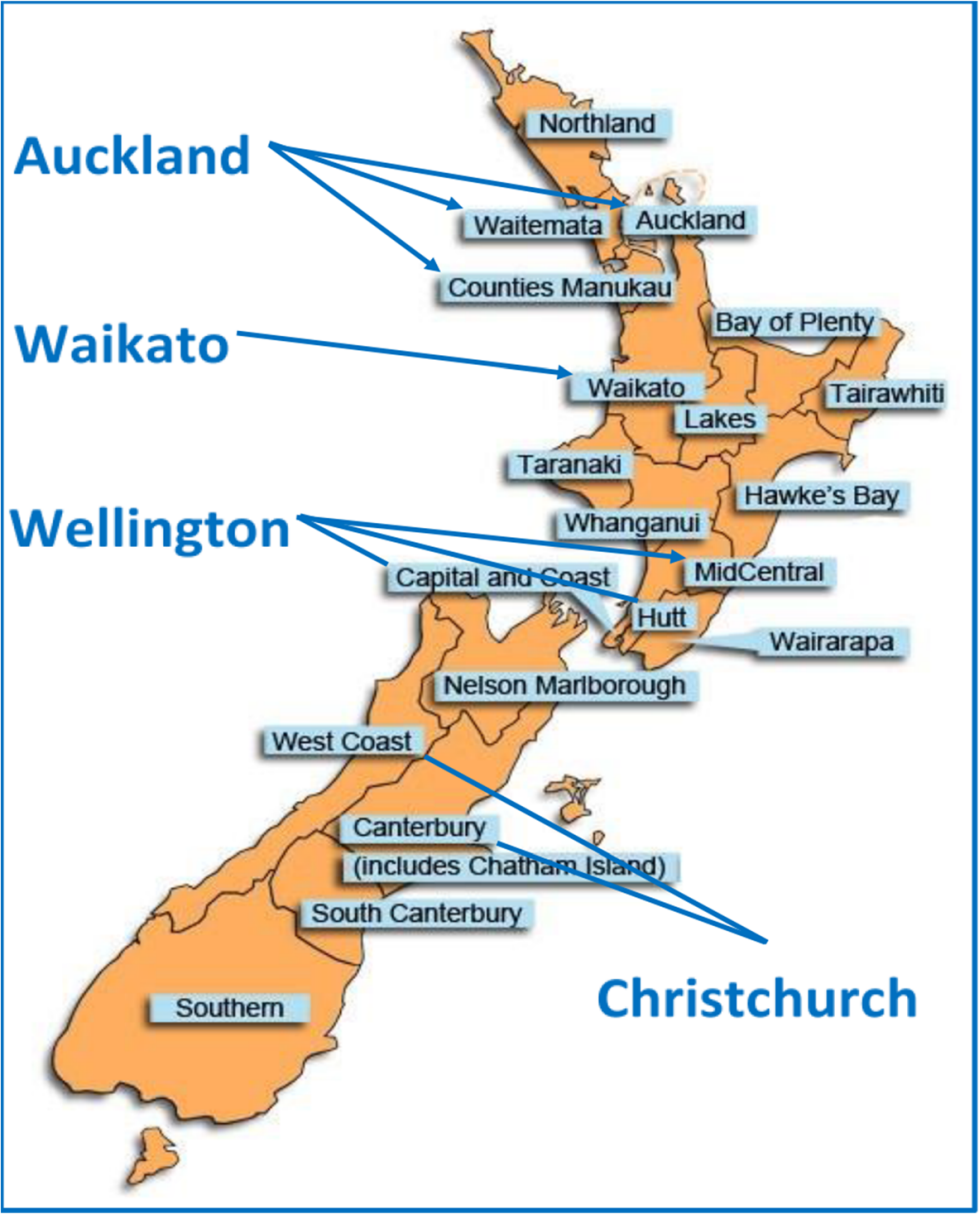
Coverage of the New Zealand Breast Cancer. Adapted from Breast Cancer Foundation. (https://www.breastcancerfoundation.org.nz)

This is an observational, retrospective cohort study of prospectively collected data, that aims to investigate by the audit of evaluating selected prognostic factors of breast cancer survival of women diagnosed with grade 3 breast cancer in New Zealand over a selected period. No other grade of breast cancer formed part of this study group. Data from the NZBCR for grade 3 breast cancer were analyzed in an attempt to stratify its impact in New Zealand. The period selected was from 1st January 2011 to 31st December 2015, which allowed a consistent overlap of data from all four registers (Fig. 1) to provide a national five-year data set from the date of diagnosis for the multiple cohorts of women.

### Data

First, we extracted data from the New Zealand Breast Cancer Register NZBCR of women diagnosed with grade 3 breast cancer. The NZBCR is made up of data collected from across the country’s four major regions. These include all patients treated for breast cancer in Auckland Region (Waitemata, Auckland, and Counties Manukau District Health Boards), Waikato, Wellington (Wairarapa, Capital & Coast, Hutt Valley District Health Boards), and Christchurch (Canterbury District Health Board), as shown in Fig. 1. These include all patients treated in public and private settings. Approximately 67% of all national New Zealand breast cancer registrations are recorded in these registers [11]. Details of the form of the NZBCR data are elaborated on https://www.breastcancerfoundation.org.nz

Sources of data for NZBCR data include pathology laboratories, oncology services, and the breast cancer registry [12]. The overall stage of diagnosis was calculated by using the staging classification of the American Joint Committee on Cancer (AJCC) 7th edition, collating data on tumor size, nodal status, and metastasis (TNM). The patients were stratified at diagnosis into stages 1–4 and X, where stage X was characterized as not assessable. The IHC HER2 status was assessed on the American Society of Clinical Oncology (ASCO) guidelines, into HER2 negative and HER2 positive. Untested status was not included, and missing data, coded as “Unknown,” were also not included in the analysis. The histological type was divided into four categories: ductal, lobular, mixed, and other. Hormone receptor status was a combination of Estrogen Receptor (ER) and Progesterone Receptor (PR) positive and negative status. This was further categorized by combining the HER2 status into molecular subtypes as luminal A (ER and /or PR +, HER2-), luminal B (ER and/or PR+, HER2-, or HER2+), Her2 enriched (ER and PR-, HER2+) and Triple Negative Breast Cancer (TNBC), (ER, PR and HER2-).

To subtype ER positive tumours: PR status, HER2 status and the Ki67 count are utilised in classifying Luminal A and Luminal B. Currently there is no consensus agreement on Ki67 reporting and cut off values amongst the New Zealand pathologists. There is inconsistent reporting in labs within the regions and nationally, resulting in incomplete data in the register, to use as a criterion, so for this study, the definition of Luminal B subtypes had to be based on hormone receptor and HER2 results.

Age at diagnosis was divided into seven groups: 20–40 years (due to the low numbers this was grouped as group 1), 41–50 years (group 2), 51–60 years (group 3), 61–70 years (group 4), 71–80 years (group 5), and > 80 (group 6). Ethnicity data were grouped into 5 categories: Māori, Pacific Islander, NZ European, Asian, and Other. Due to low numbers, the “Other” group was combined with “Asian”.

### Statistical analysis

Descriptive characterization of the study population was carried out using standard, frequency, and percentage summarization. The NZBCR characterized cause of death as breast cancer related or other causes, so univariate Fine-Gray competing risk models were carried out, instead of the traditional Cox proportional hazard regression, with Hazard Ratios (HR) reported. The reference group selected for each dependent variable was the most favorable prognostic factor outcome in each group. Cumulative Incidence Factors (CIF) are reported, as competing risk models were run. Multivariate and interaction models were not able to be run due to small subgroup cell counts. All analyses were carried out using Statistical Data Analysis (STATA) version 16.0.

## Results

### Demographic factors

Over the five-year study period from 1st January 2011 to 31st December 2015, 2493, women were diagnosed with grade 3 breast cancer, the NZBCR recorded a breast cancer fatality rate of 42.9%. The cohort’s overall demographics are summarised in Table 1, along with a descriptive summary by ethnicity. Table 2 summarises the univariate, Fine-Gray models. Briefly, women under the age of 50 were at decreased risk of five-year mortality and this difference approached statistical significance (HR: 0.57, 95% CI: 0.3–1.0, *p* = 0.06). Women greater than 70 years were at elevated risk (HR: 1.7, 95% CI, 0.9–3.0, p 0.05).; women aged 81 or greater at significantly increased risk (H: 2.2, 95% CI, 1.2–4.1, *p* < 0.05) of breast cancer-related causes. The NZ European population was at elevated risk (H: 1.6, 95% CI: 0.9–3.2, *p* = 0.11). In contrast, the Māori and Pacific Islander populations were not at elevated risk of five-year mortality (HR: 1.4, 95% CI: 0.6–3.1, *p* = 0.36) and (HR: 1.4, 95% CI: 0.5–3.6, *p* = 0.44) respectively.

**Table 1:** Demographics and characteristics of women diagnosed with grade 3 breast cancer in NZ

Overall												
**Ethnicity**	**No.**	**%**										
**Asian**	223	8.9										
**Maori**	244	9.8										
**NZ European**	1783	71.5										
**Pacific Islander**	159	6.4										
**Other**	84	3.4										
			**By Ethnicity**									
			**Asian**		**Maori**		**NZ European**		**Pacific Islander**		**Other**	
**Age**			**No.**	**%**	**No.**	**%**	**No.**	**%**	**No.**	**%**	**No.**	**%**
**< =40**	269	10.8	30	13.5	34	13.9	166	9.3	26	16.4	13	15.5
**41–50**	627	25.2	74	33.2	76	31.1	413	23.2	47	29.6	17	20.2
**51–60**	654	26.2	72	32.3	81	33.2	438	24.6	47	29.6	16	19
**61–70**	511	20.5	32	14.3	42	17.2	387	21.7	28	17.6	22	26.2
**71–80**	267	10.7	10	4.5	9	3.7	228	12.8	11	6.9	9	10.7
**> 80**	165	6.6	5	2.2	2	0.8	151	8.5	0	0	7	8.3
**Cause of Death**												
**Breast Cancer**	171	56.1	10	58.8	16	51.6	134	59.3	8	33.3	3	42.9
**Other causes**	134	43.9	7	41.2	15	48.4	92	40.7	16	66.7	4	57.1
**Histological Type**												
**Ductal**	2253	91.5	209	94.1	226	92.6	1590	90.6	149	94.3	79	94
**Lobular**	78	3.2	3	1.4	4	1.6	67	3.8	4	2.5	0	0
**Mixed**	18	0.7	2	0.9	3	1.2	13	0.7	0	0	0	0
**Other**	114	4.6	8	3.6	11	4.5	85	4.8	5	3.2	5	6
**Stage**												
**0**	5	0.2	1	0.4	0	0	3	0.2	1	0.6	0	0
**I**	709	28.4	58	26	65	26.6	537	30.1	21	13.2	28	33.3
**II**	1024	41.1	95	42.6	107	43.9	736	41.3	60	37.7	26	31
**III**	356	14.3	39	17.5	35	14.3	228	12.8	38	23.9	16	19
**IV**	363	14.6	29	13	34	13.9	251	14.1	36	22.6	13	15.5
**X**	36	1.4	1	0.4	3	1.2	28	1.6	3	1.9	1	1.2
**Subtype**												
**Luminal A**	749	41.9	63	41.7	78	42.9	526	41.3	58	48.3	24	38.1
**Luminal B**	153	8.6	16	10.6	26	14.3	99	7.8	8	6.7	4	6.3
**Her Enriched**	295	16.5	25	16.6	34	18.7	196	15.4	35	29.2	5	7.9
**Triple Negative**	592	33.1	47	31.1	44	24.2	452	35.5	19	15.8	30	47.6

**Table 2:** Univariate Fine-Gray models of women diagnosed with grade 3 breast cancer in NZ

Age	SHR	Robust SE	Z	p	CI	
41–50	0.578	0.170	−1.86	0.063	0.324	1.030
51–60	0.944	0.253	−0.22	0.830	0.558	1.597
61–70	0.728	0.213	−1.08	0.278	0.411	1.291
71–80	1.738	0.495	1.94	0.053	0.994	3.038
> 80	2.253	0.689	2.66	0.008	1.237	4.104
Ethnicity						
Maori	1.445	0.584	0.91	0.363	0.654	3.191
NZ European	1.695	0.558	1.60	0.109	0.889	3.232
Asian	1.287	0.833	0.39	0.697	0.362	4.574
Pacific Islander	1.440	0.681	0.77	0.440	0.570	3.639
Histotype						
Ductal	1.347	0.684	0.59	0.557	0.498	3.645
Mixed	0.779	0.525	−0.37	0.711	0.208	2.917
HR Status						
ER−/PR+	1.200	1.181	0.19	0.853	0.175	8.257
ER+/PR-	3.555	0.774	5.82	0.000	2.320	5.447
ER−/PR-	2.672	0.534	4.92	0.000	1.807	3.952
Stage						
II	1.829	1.081	1.02	0.307	0.574	5.828
III	1.994	1.409	0.98	0.329	0.499	7.964
IV	79.415	40.194	8.64	0.000	29.450	214.148
X	6.189	6.949	1.62	0.104	0.685	55.886
Subtype						
Luminal B	3.354	1.094	3.71	0.000	1.770	6.358
Her2 Enriched	1.659	0.519	1.62	0.105	0.899	3.062
Triple Negative	3.013	0.715	4.65	0.000	1.892	4.799

### Prognostic factors

Ductal carcinoma represented the largest proportion of grade 3 cancers (Table 1). There was no statistically significant difference in the five–year mortality by histological type (Table 2). The histological subtype Luminal A was documented in the largest proportion of women with grade 3 breast cancer followed by TNBC (Table 1). HER2 enriched were at a higher, but not significant, risk of five-year mortality (HR: 1.6, 95% CI: 0.8–3.0, *p* = 0.10) when compared to luminal A and TNBC that were higher and significant risk of five-year mortality (HR: 3.0, 95% CI: 1.8–4.7, *p* < 0.00).

The ER/PR positive tumors were the most common hormone receptor status in women with grade 3 breast cancer (Table 1). Women with PR-negative combinations were at increased risk of five-year mortality ER+/PR- (HR: 3.5, 95% CI: 2.3–5.7, *p* = 0.00) and ER−/PR- (HR: 2.6, 95% CI: 1.8–3.9, *p <* 0.00). Of note, the Pacific Islander group represented more ER/PR positive tumors whereas the Māori group represented more ER/PR negative (Table 1).

A small percentage of women were excluded due to results not captured (Table 1). Early-stage disease: stage I and stage II predominated at diagnosis, 27.18, 40.08% respectively (Table 1). Comparison between the two did not yield statistically significant differences in five-year mortality. Stage III cancers exhibited a statistically significant, increased risk; however, the error in these findings is large. This is due to the inconsistent recording of the stage at diagnosis so our confidence in this particular result is preliminary.

**Fig. 2:**
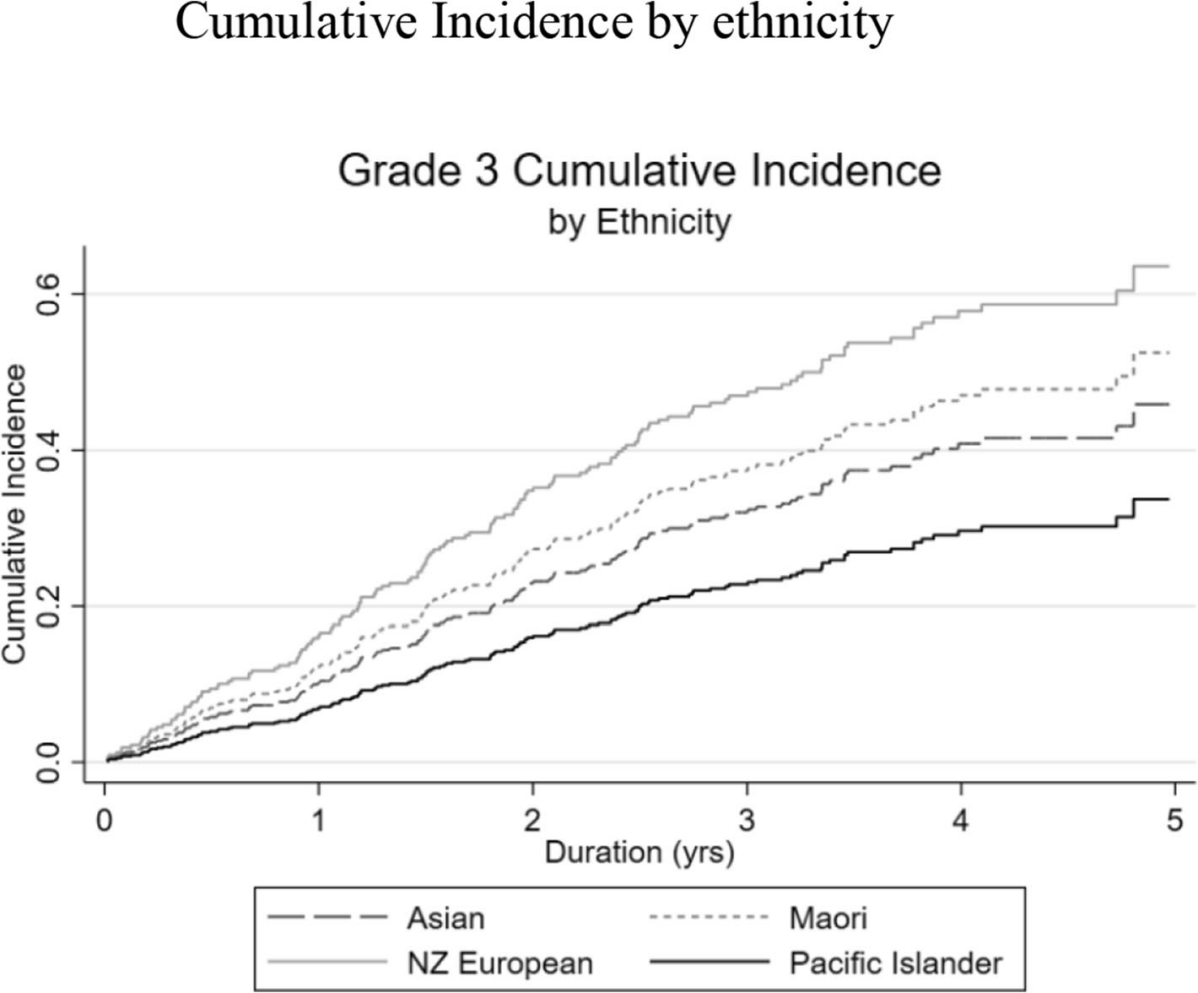
Cumulative Incidence of five-year mortality of Grade 3 breast cancer by ethnicity

**Fig. 3:**
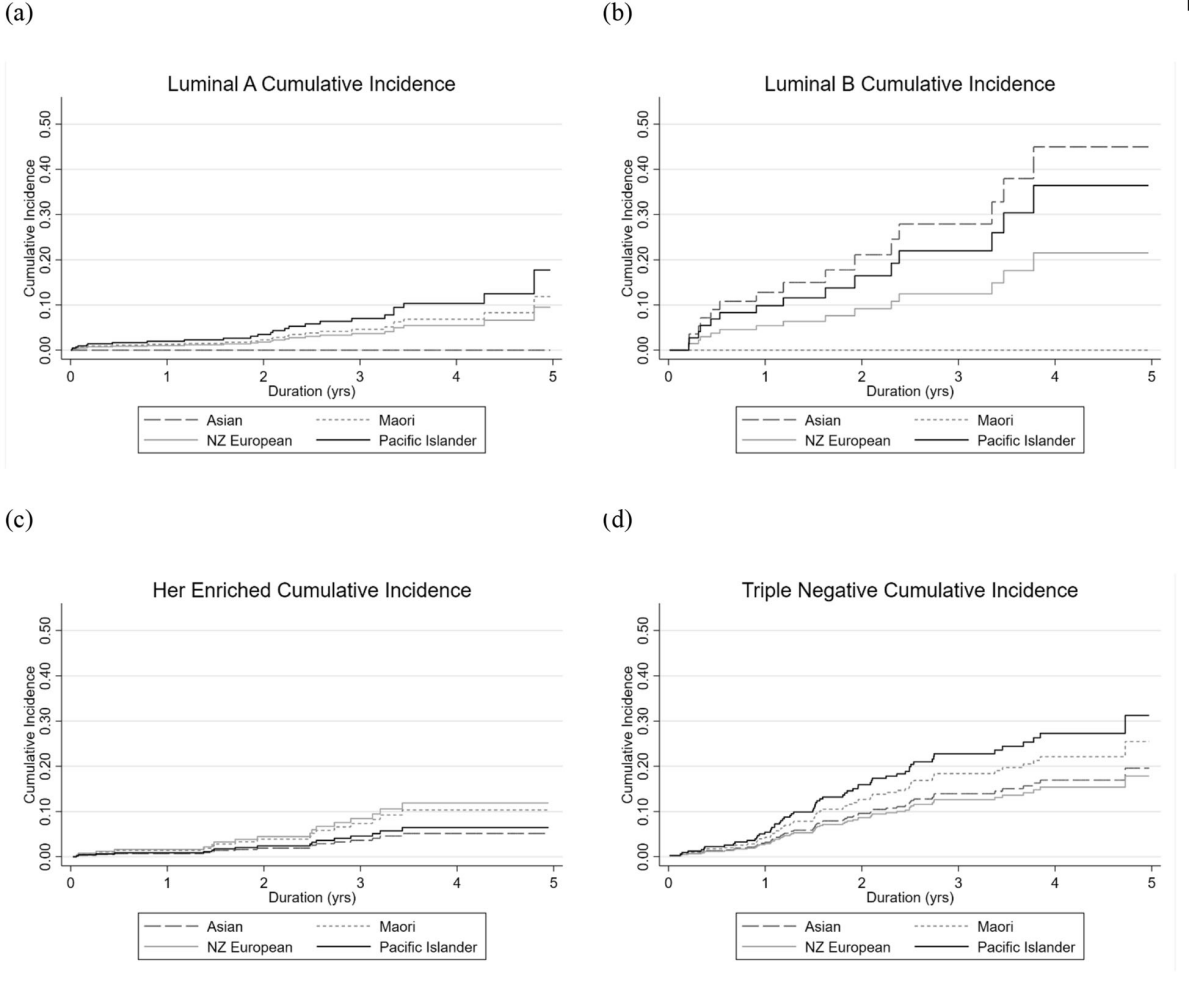
a. Cumulative Incidence of five-year mortality of molecular subtype Luminal A by Ethnicity. b. Cumulative Incidence of five-year mortality of molecular subtype Luminal B by ethnicity. c. Cumulative Incidence of five-year mortality of molecular subtype Her2 enriched by Ethnicity. d. Cumulative Incidence of five-year mortality of molecular subtype TNBC by Ethnicity

Figures 2 and 3 summarize the cumulative incidence factors (CIF) by ethnicity alone and by molecular subtype and ethnicity respectively. Of note, Pacific Islanders had the lowest overall five-year CIF (Fig. 2). In contrast, this same community experienced the highest five-year CIF for TNBC (Fig. 3d). It should be noted from Table 1 that 48% of grade 3 cancers in the Pacific Islander group were luminal A subtype. This subtype has the lowest risk of five-year mortality cancer. The percentage of Pacific Islanders with TNBC is 15.8%. In contrast, for all other defined ethnic groups, there was a tendency for relatively lower proportions of luminal A (range: ~ 41%) and higher percentages of TNBC (range: 24–36%). Multivariate, Fine-Gray models were not able to be run due to low subgroup breast cancer mortality-ethnicity cell counts.

## Discussion

From this study, we found that survival rates for women diagnosed with grade 3 breast cancer varied across the selected prognostic factors and confirms that it is dependent on multiple factors. Despite the high grade, the outcome for some women is not poor.

A limited range of common prognostic factors deemed relevant for this study was selected, to provide a preliminary understanding of the characteristics of grade 3 breast cancer in New Zealand women.

The mortality is related to breast cancer only. NZ subjects in the older age group of > 70 years were at increased risk of five-year mortality. This is consistent with the literature finding [13–21] where these studies found that women in the older age group diagnosed with breast cancer had poor outcomes when compared to women in the younger age group.

Amongst all ethnicities, the New Zealand European population was at elevated risk overall; however, the CIF across ethnicity varied by molecular subtype The analysis also reported that NZ European group presented the largest group in proportion to the population for HER2 enriched and TNBC and the subjects from these two molecular subtypes were at increased risk of five-year mortality. Whilst the morphology type showed there was no statistically significant difference observed, the molecular subtypes (HER2 enriched and TNBC) subjects were at increased risk of five-year mortality. The latter is consistent with literature findings [22–30] where these studies found that Her2 expression was a strong predictor for poor outcome in women with breast cancer. This study could not do full SHR’s for the ER and PR for the various ethnicity groups due to small cell counts, so our outcome is preliminary here.

Luminal B subtype has a poorer prognosis and women with PR-negative combinations (luminal B subtype) were at increased risk of five-year mortality and this is consistent with literature finding [31] where the study found that the luminal B subtype had pathological and clinical features that showed poor response for treatment with poorer outcome. It must be noted that in this study the luminal B subtype was relatively more frequent in the Maori group. In contrast, the Luminal A subtype is associated with better five-year survival. However, despite, showing a lower and a better biological profile of tumor risk marker, overall, the Pacific Islander group showed poorer survival in comparison with the other ethnic groups in this study.

Studies describing equity-focussed improvements in health care may have improved the survival disparity between Māori and NZ European women [9, 10, 32, 33]. The studies highlight that when there is improvement in service access, quality, and timeliness of care, patient risk profiles, and understanding of biological factors, there is the opportunity for earlier intervention and therefore improved survival [9, 10]. However, whilst studies have reported on ethnic inequities in breast cancer outcomes in New Zealand, there is insufficient data to fully understand its underlying contribution to these differences [9, 10, 34].

From the analysis, we found that survival rates for breast cancer varied across the selected prognostic factors and confirms that it is dependent on multiple factors. Previous studies have shown that some of these include: patient factors, tumor biology, and ethnicity, as well as access to health interventions and treatment, socioeconomic status, availability of drugs, and the treatment type available [35–37]. These studies showed that survival rates varied across prognostic factors. However, it is beyond the scope of this study to be able to address the ethnic and socioeconomic status and its impact on the overall survival of women with grade 3 breast cancer.

Based on the literature and results, to help understand survival disparities within the ethnic groups, in particular Maori and Pacific Islander, the following recommendations/suggestions should be considered: understanding tumor biology and genetic susceptibility of grade 3 breast cancer, understanding of patient risk profile, understanding how better access to service impacts on the outcome, quality and timeliness of care for patients, understanding mortality with family history, benefits of personalized care.

Further analysis of the prognostic factors that were not included in this study such as lymph-vascular invasion (LVI), height, weight, biomarker (FISH) studies, number of nodes removed, type of surgery, type of treatment, and loco-regional recurrence status should be included.

These factors may each independently and/or collaboratively influence survival and could help to further categorize grade 3 breast cancer in NZ women.

### Strengths and limitations of this study

This is the first retrospective study using prospectively collected data from the NZBCR to analyze grade 3 breast cancers in NZ as a stand-alone grade, representing a study strength.

The registry represents 67% of all breast cancer diagnosed in New Zealand). Data analysis was used to assess the impact of the disease in New Zealand with an emphasis on grade 3 breast cancer. The data is linked to census data to allow researchers to investigate the outcome of breast cancer across the different regions. The study also lies within the population-based nature of the breast cancer registry and the outcome (death) is linked via the patient record. Furthermore, the various ethnic groups present in the four regions are included reflective of the population of New Zealand.

The study makes an initial contribution to the understanding of high-grade malignancy and has broad application in understanding survival rates of women with grade 3 breast cancer in New Zealand. This including other prognostic factors will give a better understanding of the survival differences with this high-grade malignancy.

The data that we used also has some limitations. The histologic categorizations used that was based on diagnoses made by multiple pathologists in multiple institutions. The diagnostic criteria may vary somewhat by both individual pathologists and establishment, resulting in a certain degree of misclassification error.

Other limitations in our data were mainly in the form of missing values. There is dependence on the data administrators to enter all data accurately and consistently on time. The data standardization for all four registries in NZ took effect from 2017 making future studies using the NZBCR more efficient. The data standardization will allow for missing data to be taken into consideration and will enable us to include all this missing information in an appropriate way to improve the overall picture for future studies using the NZBCR.

There is limited literature on breast cancer for Pacific Island women. Studies thus far have reported that Pacific Island women have lower breast cancer incidence but higher mortality risk than Māori and European women in New Zealand [38], which appears to be re-confirmed in this study. Therefore, this study reflects a strength in that it provides additional information on the Pacific Islander group with grade 3 breast cancer.

The inconsistent recording of disease stage at diagnosis and treatment data may pose significant limitations for researching the causes of inequities [32]. Whilst stage III breast cancers exhibited a statistically significant increased risk of five-year mortality, due to the inconsistent recording, the error in these findings are large, therefore our confidence in the results for this prognostic factor (stage at diagnosis) is tentative.

The histologic type of breast cancer in this group was assigned by multiple pathologists in multiple institutions. Although standardized guidelines from the Nottingham grading system are used, there is subjective variation, therefore the diagnostic criteria may vary somewhat by both individual pathologists and establishment, resulting in a certain degree of misclassification error.

Furthermore, this study did not include data on health insurance status or lifestyle factors (e.g., body mass index, weight, physical activity, diet, etc.,) breast density, or genetic testing, all of which can influence breast cancer outcome since these were not available from the registries [39–41].

Finally, whilst studies have reported on ethnic inequities in breast cancer outcomes in New Zealand, there is insufficient data to fully understand the underlying causes of these differences [35, 37, 42].

## Conclusion

Grade 3 breast cancer is referred to as heterogeneous and high-grade cancer. Despite the high-grade cancer, we found that five-year survival varies by a combination of biological and ethnicity factors. Women in the “Asian” ethnicity group with luminal A subtype, presented with the best prognosis. The New Zealand Europeans, Maori and the Pacific Islanders are at increased risk of early death. Pacific Islanders with Luminal A or TNBC are at the greatest risk. The trajectory towards poor overall survival for Pacific Islanders needs more research to identify the causes of the survival disparity. A multitude of other factors may each independently or collaboratively also influence survival. Elucidation of these factors may help to further categorize grade 3 breast cancer and contribute to a greater understanding of the risk factors of grade 3 breast cancer in NZ, and possibly enable better outcomes.

## Data Availability

The data that support the findings of this study are available from New Zealand Breast Cancer Register but restrictions apply to the availability of these data, which were used under license for the current study, and so are not publicly available. Data are however available from the authors upon reasonable request and with permission of the New Zealand Breast Cancer Register.

## Abbreviations

AUTEC: Auckland University of Technology Ethics Committee
CIF: Cumulative Incidence factor
ER: Estrogen Receptor
HER2: Human Epidermal Growth Factor Receptor 2
IHC: Immunohistochemistry
NZ: New Zealand
NZBCR: New Zealand Breast Cancer Register
PR: Progesterone Receptor
HR: Hazard Ratio
STATA: Statistical Data Analysis
TNBC: Triple-Negative Breast Cancer
TNM: Tumour, Node, Metastasis
